# A Descriptive Analysis of Nutrient Density and Nutritional Value of Meat Products Using the Canadian Nutrient File Database

**DOI:** 10.3390/foods15152612

**Published:** 2026-07-26

**Authors:** Benjamin M. Bohrer

**Affiliations:** Department of Animal Sciences, The Ohio State University, Columbus, OH 43210, USA; bohrer.13@osu.edu; Tel.: +1-937-402-8536

**Keywords:** protein, nutrient density, nutritional value, meat products, non-meat foods

## Abstract

The purpose of this project was to investigate the nutrient density and nutritional value of meat products, seafood products, and plant-derived protein food products using the 2015 Canadian Nutrient File database. Particular emphasis was placed on comparing nutrient density before and after cooking or preparation, as well as evaluating the cost of foods relative to the nutrients they provide using Canadian retail prices collected over a five-month period. Overall, meat and seafood products were consistently rich sources of protein and key micronutrients, including zinc and vitamin B12, whereas many plant-derived protein foods, including kale, broccoli, spinach, lentils, beans, and quinoa, contained substantially lower protein concentrations on both an uncooked (as purchased) and cooked (as prepared) basis. Although the concentrations of nutrients such as fat, iron, phosphorus, and sodium varied among food products, these findings demonstrate meaningful differences in nutrient density and nutrient cost across protein food categories. These results provide a useful framework for consumers, health professionals, and policymakers when evaluating nutrient-rich protein food choices and underscore the importance of considering both nutrient composition and economic value in dietary recommendations. Future research should extend these comparisons by incorporating direct analytical measurements of foods, assessments of protein quality and indispensable amino acid digestibility, mineral bioavailability, and the effects of food processing and preparation on nutrient utilization to better characterize the nutritional contributions of diverse protein food sources.

## 1. Introduction

In most countries of the world, the consumption of meat and seafood products are held in high esteem and are regarded as food products with high nutritional value [1,2]. Nutritionally, meat and meat products are considered an excellent source of zinc, heme-iron, bio-available B vitamins, and essential amino acids [3,4]. However, in developed countries there has been a lost appreciation for the nutritional benefits of meat and seafood products despite the support that meat and seafood consumption significantly contributes to a healthy and well-balanced diet and lifestyle. There has been a growing tendency for individuals to question the nutritional profile of meat products, and particularly the healthiness of red meat consumption. With recent reports published by various interest groups, national media outlets, and even the scientific community, the suggestion to reduce the consumption of meat is becoming a persistent battle for the meat industry. Specific to red meat (i.e., beef and pork), health concerns are undoubtedly responsible for the slight decline in per capita consumption of beef and pork in developed countries since 1980 [5,6]. These changes in consumer preferences are also reflected in global meat consumption patterns. For example, global per capita consumption of chicken has increased substantially over recent decades while global per capita consumption of beef has been relatively flat. This increase reflects a broader global shift toward poultry as a preferred protein source, and chicken is now one of the most widely consumed meats worldwide [7].

The percentage of individuals choosing not to consume meat for non-religious reasons is a relatively small percentage of people (estimated 2–10% in developed nations) [8]. However, this portion of the population still makes up a significant population of people around the world, and this demographic of people has a significant influence on the food marketplace. The growth of non-meat consumers was further validated by the 2014 internet chatter measurements (number of mentions on the internet) that ranked the dietary trend vegan as number one (591% growth in mentions) and dietary trend vegetarian as number three (152% growth in mentions) of all dietary trends evaluated [9]. This trend has continued with increased consumer interest in plant-derived protein products and alternative protein sources [10,11]. There have been many studies that evaluate and discuss the health benefits and challenges of a well-planned vegetarian or vegan diet [12,13]. However, it is important to consider and review the nutritional content of the food products that are deemed to be high sources of dietary protein.

Of all purchasing decisions that go through a consumer’s mind (i.e., health, emotions, convenience, tradition/culture), the price of meat versus other protein sources is often a decisive factor [14]. Consumers typically believe meat is more expensive than other protein sources. A recent research review that focused on comparing nutrient density and nutritional value of meat products and plant-derived protein foods, discovered that if nutrients were fully considered on a cost-per-nutrient basis for meat products and plant-derived protein foods like legumes, nuts, beans, and other plant-derived foods, then cost of nutrients (proteins, minerals, and vitamins when expressed on a per-dollar basis) were very similar in price [15]. If diversity of amino acids and bio-availability of amino acids, minerals, and vitamins were fully considered, meat products would likely be cheaper (expressed on a per nutrient digested basis) when compared with plant-derived foods.

Several scientific reviews and descriptive analyses have independently summarized the nutritional content of meat products [16,17], seafood products [18,19], and crop/legume products [20,21]. However, this literature is scattered, and more comprehensive analyses are necessary to fully compare nutritional content of meat products with plant-derived products deemed high in protein. Furthermore, greater awareness of interactive, tabular documents that can be accessed by the consumer for the analysis of the cost of nutrients and ratio of different nutrients should be prioritized by governing bodies. Also, no data have been exclusive to Canada (i.e., using a Canadian nutritional database and using Canadian food prices). Therefore, the overall objective of this project was to investigate the nutrient density and cost of nutrients for meat products and plant-derived protein foods using the Canadian Nutrient File (CNF) database and Canadian retail food prices.

## 2. Methodology

### 2.1. Data Collection

The methodology of this study generally followed that of Bohrer [15] and Huang et al. [22] with a few exceptions. This research project placed particular emphasis on the comparison of meat products (beef, pork, lamb, and poultry), seafood products, and plant-derived protein food products. Specific information included nutrient density calculated per weight, nutrient density calculated per calorie, and nutrient density calculated per retail cost. In an effort to provide consumers with the ability to weigh the value of foods when constructing a well-balanced diet, ratios of these nutrients were available in both the uncooked (as purchased) and cooked (as prepared) form within the created database.

Nutrient composition data were obtained exclusively from the 2015 Canadian Nutrient File (CNF) database [23]. A single version of the CNF was used throughout the study to ensure consistency in nutrient composition values across all food products and to avoid variability associated with combining data from multiple database versions. Food items were selected based on the closest match to the retail products with respect to product type, formulation, and preparation state. The selection of plant-derived foods primarily represented whole-food sources and did not include several commercially important plant-derived protein products, such as tofu, tempeh, seitan, textured vegetable protein, soy protein isolates, and newer plant-derived meat alternatives. These products may have substantially different protein concentrations, amino acid profiles, fortification strategies, and nutrient densities compared with minimally processed plant foods. Therefore, the results of this study should not be interpreted as representing all plant-derived protein foods but rather the specific foods included in this analysis. In total, the nutritional composition of 25 beef products, 15 pork products, 6 lamb products, 6 chicken products, 6 turkey products, 11 fish products, and 15 plant-derived protein products were indexed and reported. Both uncooked (as purchased) and cooked (as prepared) forms were evaluated. Nutrients were based on those available on the database and included energy value (calories), protein, fat, saturated fat, cholesterol, vitamin B12, iron, phosphorus, zinc, and sodium.

The average retail cost of the products was assessed for uncooked (as purchased) products over 5 months from December 2019 to April 2020. One day was chosen in the middle of each month and a retail assessment was conducted. The retail assessment consisted of reporting the cost of products (as available) on a 100-g basis from three different retail outlets in southwestern Ontario. The average cost of products was then averaged among the three retailers and then among the five months. All retail costs were reported in Canadian currency. Retail prices were obtained for products in their uncooked (as-purchased) form. Because many of the products evaluated are not commercially available in their cooked (as-prepared) form, the average retail cost of the uncooked product was also used to represent the retail cost of the cooked product for comparative calculations. In other words, no additional cooking-related costs or yield-adjusted retail prices were applied; the same purchase price per unit weight was assigned to both the uncooked and cooked datasets, under the assumption that consumers purchase these products raw and prepare it themselves.

Data were presented in tables and consisted of the nutrient composition of uncooked (as purchased) and cooked (as prepared) food products using the 2015 CNF database, and the cost of nutrients for uncooked (as purchased) and cooked (as prepared) food products using a Canadian retail assessment and the 2015 CNF database.

### 2.2. Limitations

A limitation of this review is the reliance on nutrient composition data from the 2015 CNF rather than direct analytical measurements of the specific products evaluated. Although the CNF provides standardized and nationally representative nutrient values, nutrient composition may vary among commercially available products due to differences in cultivar or breed, genetics, agricultural production practices, geographic origin, processing, formulation, and seasonal variation. To improve comparability, food items were selected based on the closest match to the retail products in terms of product type, formulation, and preparation state, and all nutrient values were obtained from the same standardized database. Consequently, the results should be interpreted as comparisons of representative food categories rather than exact nutrient profiles of individual commercial products.

The nutrient cost analysis should be interpreted within the context of several limitations associated with retail price estimation. Food prices vary due to geographic location, retailer, seasonality, promotional pricing, package size, and consumer purchasing patterns. Additionally, this analysis did not account for edible yield differences, including trimming losses, preparation losses, or cooking-associated weight changes, which may influence the final cost per unit of nutrient available for consumption. Therefore, the reported nutrient costs represent comparative estimates based on observed retail prices rather than universal consumer costs. Future studies incorporating larger geographic sampling, longitudinal price monitoring, and edible yield adjustments would provide a more comprehensive assessment of the economic value of different protein foods.

Furthermore, this project was designed as a descriptive comparison of nutrient density and nutrient cost among representative protein foods. Thus, inferential statistical analyses were not conducted. Mean values and standard deviations were calculated to describe variation within each food category. Therefore, comparisons presented throughout the manuscript represent observed differences among evaluated food products and should not be interpreted as statistically significant differences.

## 3. Results and Discussion

### 3.1. Nutrient Density for Uncooked and Cooked Food Products

Nutrient density (energy, protein, fat, saturated fat, cholesterol, vitamin B12, iron, phosphorus, zinc, sodium, protein:calories, protein:total fat, protein:saturated fat, and saturated fat:total fat) using the 2015 CNF database were presented for uncooked/unprepared foods (as purchased) and for cooked (as prepared) products.

#### 3.1.1. Dietary Energy

Dietary energy of the food products in their uncooked and cooked/prepared state are presented in Table S1. The dietary energy content of uncooked (as purchased) beef, pork, poultry, and seafood products was an average of 169 ± 61 kcal/100 g serving and values ranged from 74 kcal/100 g serving (haddock) to 417 kcal/100 g serving (pork bacon). The dietary energy content of uncooked (as purchased) plant-derived products was an average of 232 ± 218 kcal/100 g serving and values ranged from 23 kcal/100 g serving (spinach) to 579 kcal/100 g serving (almonds).

The dietary energy content of cooked (as prepared) beef, pork, poultry, and seafood products was an average of 221 ± 67 kcal/100 g serving and values ranged from 90 kcal/100 g serving (haddock) to 548 kcal/100 g serving (pork bacon). The dietary energy content of cooked (as prepared) plant-derived products was an average of 184 ± 222 kcal/100 g serving and values ranged from 22 kcal/100 g serving (pinto beans) to 598 kcal/100 g serving (almonds).

In general, plant-derived products had a greater range in value for dietary energy content compared with beef, pork, poultry, and seafood products—with green vegetables (kale, broccoli, spinach) having very low dietary energy content, whereas nuts (peanuts, almonds, cashews) have much higher dietary energy. Dietary energy, or calories, is often considered by consumers throughout the world. With that said—it is uncommon for people in developed and even underdeveloped nations to have deficiencies in energy intake, and most people in developed nations are actually more concerned with monitoring their intake of dietary energy for weight loss or weight maintenance. Excess intake of dietary energy is stored in the body as fat, which causes obesity and a host of related health problems [24,25].

#### 3.1.2. Dietary Protein

Dietary protein of the food products in their uncooked and cooked/prepared state are presented in Table S2. The dietary protein content of uncooked (as purchased) beef, pork, poultry, and seafood products was an average of 19.77 ± 2.60 g/100 g serving and values ranged from 11.80 g/100 g serving (chicken egg) to 24.40 g/100 g serving (yellowfin tuna). The dietary protein content of uncooked (as purchased) plant-derived products was an average of 11.67 ± 8.40 g/100 g serving and values ranged from 2.82 g/100 g serving (broccoli) to 25.80 g/100 g serving (peanuts).

The dietary protein content of cooked (as prepared) beef, pork, poultry, and seafood products was an average of 28.35 ± 5.32 g/100 g serving and values ranged from 11.80 g/100 g serving (chicken egg) to 40.38 g/100 g serving (beef eye of round steak). The dietary protein content of cooked (as prepared) plant-derived products was an average of 8.37 ± 7.08 g/100 g serving and values ranged from 1.86 g/100 g serving (pinto beans) to 24.35 g/100 g serving (peanuts).

Adequate dietary protein is important for individuals during all stages of life, and in particular individuals must meet the requirements for essential or indispensable amino acids (phenylalanine, valine, threonine, tryptophan, isoleucine, methionine, histidine, leucine, and lysine) in the diet [26,27]. When the requirements for individual essential amino acids are not met in the diet, many physical and biochemical issues can occur [28]. Although this review focused primarily on protein concentration and nutrient density rather than amino acid composition or protein quality, the nutritional value of a protein source is also influenced by its indispensable amino acid profile, digestibility, and bioavailability.

Previous research has demonstrated that animal-derived proteins generally contain a more balanced profile of indispensable amino acids and consistently provide adequate levels of amino acids required for human metabolism compared with many individual plant-derived protein sources [22]. In contrast, many plant-derived proteins may be limited by one or more indispensable amino acids, such as lysine in cereal grains or methionine in legumes, although complementary combinations of plant-derived proteins can improve overall amino acid adequacy. Additionally, differences in protein structure, processing methods, and the presence of anti-nutritional compounds, including phytates, tannins, and other phenolic compounds, may influence amino acid and protein digestibility.

Protein quality assessment methods have evolved to incorporate both amino acid composition and digestibility. The Protein Digestibility Corrected Amino Acid Score (PDCAAS) evaluates protein quality based on the limiting indispensable amino acid relative to a reference amino acid pattern and adjusts this value based on overall protein digestibility. However, PDCAAS has limitations, including truncation of scores above 1.0 and the use of fecal rather than ileal digestibility measurements. The Digestible Indispensable Amino Acid Score (DIAAS) was developed as an alternative approach that evaluates the digestibility of individual indispensable amino acids at the end of the small intestine and is considered a more accurate representation of amino acid availability for human metabolism. Using these approaches, many animal-derived proteins, including meat, poultry, seafood, eggs, and dairy products, have demonstrated high protein quality due to their favorable indispensable amino acid profiles and high digestibility. However, certain plant-derived proteins can also provide high-quality protein, particularly when processed to improve digestibility or consumed in combinations that complement amino acid limitations.

The findings from this descriptive analysis demonstrate that, on an uncooked basis, beef, pork, poultry, and seafood products provided greater and more consistent protein concentrations compared with the plant-derived whole foods evaluated (approximately 41% greater protein concentration on an uncooked basis and 70% greater protein concentration on a cooked basis). However, these comparisons represent differences in protein quantity and nutrient composition rather than complete assessments of protein nutritional quality. Future evaluations of protein foods should incorporate measures of amino acid composition, DIAAS or other digestibility-adjusted protein quality metrics, and nutrient bioavailability to provide a more comprehensive assessment of their contribution to human nutrition.

#### 3.1.3. Total Fat, Saturated Fat, and Cholesterol

Total fat, saturated fat, and cholesterol of the food products in their uncooked and cooked/prepared state are presented in Table S3. The total fat content of uncooked (as purchased) beef, pork, poultry, and seafood products was an average of 9.36 ± 7.37 g/100 g serving, and values ranged from 0.45 g/100 g serving (haddock) to 39.69 g/100 g serving (cured pork bacon). The total fat content of uncooked (as purchased) plant-derived products was an average of 11.18 ± 19.87 g/100 g serving, and values ranged from 0.37 g/100 g serving (broccoli) to 49.93 g/100 g serving (almonds).

The total fat content of cooked (as prepared) beef, pork, poultry, and seafood products was an average of 11.08 ± 7.32 g/100 g serving, and values ranged from 0.55 g/100 g serving (haddock) to 43.27 g/100 g serving (cured pork bacon). The total fat content of cooked (as prepared) plant-derived products was an average of 11.03 ± 20.90 g/100 g serving, and values ranged from 0.22 g/100 g serving (green peas) to 52.54 g/100 g serving (almonds).

The saturated fat content of uncooked (as purchased) beef, pork, poultry, and seafood products was an average of 3.28 ± 2.70 g/100 g serving, and values ranged from 0.09 g/100 g serving (haddock) to 13.30 g/100 g serving (cured pork bacon). The saturated fat content of uncooked (as purchased) plant-derived products was an average of 1.43 ± 2.58 g/100 g serving, and values ranged from 0.04 g/100 g serving (broccoli) to 7.78 g/100 g serving (cashews).

The saturated fat content of cooked (as prepared) beef, pork, poultry, and seafood products was an average of 4.02 ± 2.66 g/100 g serving, and values ranged from 0.11 g/100 g serving (haddock) to 14.19 g/100 g serving (cured pork bacon). The saturated fat content of cooked (as prepared) plant-derived products was an average of 1.57 ± 3.11 g/100 g serving, and values ranged from 0.04 g/100 g serving (spinach) to 9.16 g/100 g serving (cashews).

Cholesterol content of uncooked (as purchased) beef, pork, poultry, and seafood products was an average of 68 ± 39 mg/100 g serving and values ranged from 39 g/100 mg serving (yellowfin tuna) to 366 mg/100 g serving (chicken egg). Cholesterol content of uncooked (as purchased) plant-derived products was zero as cholesterol is only found in animal-derived foods.

Cholesterol content of cooked (as prepared) beef, pork, poultry, and seafood products was an average of 89 ± 40 mg/100 g serving and values ranged from 47 g/100 mg serving (yellowfin tuna) to 366 mg/100 g serving (chicken egg). Cholesterol content of cooked (as prepared) plant-derived products was zero as cholesterol is only found in animal-derived foods.

In recent years, consumers are becoming more informed and more critical of the food they consume. Of these concerns, they are extremely critical of the quality and quantity of fat in their diet [29]. The correlation between fats in animal-derived products and health complications, namely cardiovascular disease, has been studied, and recommendations include limiting fats from animal-derived products [30,31]. When evaluating the quality of fats in the diet, the two most characteristic effects of fats in animal-derived products is the increased amount of saturated fats and the presence of cholesterol compared to fats in plant-derived products [32]. Generally, the total fat content of animal-derived food products and plant-derived food products is highly variable (i.e., there are low-fat and high-fat options in both categories). However, saturated fat content was greater in animal-derived food products compared with plant-derived foods. This should be a consideration for consumers when selecting which protein foods to consume.

#### 3.1.4. Vitamin B12

Vitamin B12 of the food products in their uncooked and cooked/prepared state are presented in Table S4. Vitamin B12 content of uncooked (as purchased) beef, pork, poultry, and seafood products were an average of 1.70 ± 1.12 µg/100 g serving and values ranged from 0.25 µg/100 g serving (chicken wing) to 7.79 µg/100 g serving (trout). Vitamin B12 content of uncooked (as purchased) plant-derived products was zero as Vitamin B12 is only found in animal-derived foods.

Vitamin B12 content of cooked (as prepared) beef, pork, poultry, and seafood products was an average of 1.96 ± 1.16 µg/100 g serving and values ranged from 0.27 µg/100 g serving (chicken back) to 7.49 µg/100 g serving (trout). Vitamin B12 content of cooked (as prepared) plant-derived products was zero as naturally occurring Vitamin B12 is found almost exclusively in animal-derived foods.

Vitamin B12 is an important water-soluble vitamin required for proper red blood cell formation, neurological function, and DNA synthesis. It goes without saying that individuals not consuming animal-derived foods are at risk of vitamin B12 deficiency. The Food and Nutrition Board [33] recommends that adults consume 2.0 mcg/d of vitamin B12.

It is possible for individuals not consuming animal-derived foods to eat foods with fortified vitamin B12, or to consume dietary vitamin B12 supplements in an over-the-counter pharmaceutical form [34].

#### 3.1.5. Mineral Content (Iron, Phosphorus, Zinc, Sodium)

Mineral content of the food products in their uncooked and cooked/prepared state are presented in Table S5. The iron content of uncooked (as purchased) beef, pork, poultry, and seafood products was an average of 1.21 ± 0.69 mg/100 g serving and values ranged from 0.16 mg/100 g serving (halibut) to 2.75 mg/100 g serving (beef tenderloin). The iron content of uncooked (as purchased) plant-derived products was an average of 3.25 ± 1.84 mg/100 g serving, and values ranged from 0.73 mg/100 g serving (broccoli) to 6.68 mg/100 g serving (cashews).

The iron content of cooked (as prepared) beef, pork, poultry, and seafood products was an average of 1.78 ± 1.11 mg/100 g serving and values ranged from 0.20 mg/100 g serving (halibut) to 4.40 mg/100 g serving (beef bottom round steak). The iron content of cooked (as prepared) plant-derived products was an average of 2.20 ± 1.49 mg/100 g serving, and values ranged from 0.66 mg/100 g serving (pinto beans) to 6.00 mg/100 g serving (cashews).

Phosphorus content of uncooked (as purchased) beef, pork, poultry, and seafood products was an average of 189 ± 30 mg/100 g serving and values ranged from 113 mg/100 g serving (chicken back) to 278 mg/100 g serving (yellowfin tuna). Phosphorus content of uncooked (as purchased) plant-derived products was an average of 247 ± 194 mg/100 g serving, and values ranged from 37 mg/100 g serving (kidney beans) to 593 mg/100 g serving (cashews).

Phosphorus content of cooked (as prepared) beef, pork, poultry, and seafood products was an average of 226 ± 56 mg/100 g serving and values ranged from 101 mg/100 g serving (cured pork ham) to 506 mg/100 g serving (cured pork bacon). Phosphorus content of cooked (as prepared) plant-derived products was an average of 171 ± 156 mg/100 g serving, and values ranged from 28 mg/100 g serving (kale) to 490 mg/100 g serving (cashews).

The zinc content of uncooked (as purchased) beef, pork, poultry, and seafood products was an average of 2.88 ± 1.83 mg/100 g serving and values ranged from 0.32 mg/100 g serving (haddock) to 7.07 mg/100 g serving (beef short ribs). The zinc content of uncooked (as purchased) plant-derived products was an average of 1.94 ± 1.64 mg/100 g serving, and values ranged from 0.40 mg/100 g serving (kidney beans) to 5.78 mg/100 g serving (cashews).

The zinc content of cooked (as prepared) beef, pork, poultry, and seafood products was an average of 4.33 ± 2.99 mg/100 g serving and values ranged from 0.40 mg/100 g serving (haddock) to 11.46 mg/100 g serving (beef chuck blade roast). The zinc content of cooked (as prepared) plant-derived products was an average of 1.46 ± 1.50 mg/100 g serving, and values ranged from 0.17 mg/100 g serving (pinto beans) to 5.60 mg/100 g serving (cashews).

The sodium content of uncooked (as purchased) beef, pork, poultry, and seafood products was an average of 91 ± 142 mg/100 g serving and values ranged from 43 mg/100 g serving (catfish) to 1088 mg/100 g serving (cured pork ham). The sodium content of uncooked (as purchased) plant-derived products was an average of 28 ± 42 mg/100 g serving, and values ranged from 1 mg/100 g serving (almonds) to 153 mg/100 g serving (pinto beans).

The sodium content of cooked (as prepared) beef, pork, poultry, and seafood products was an average of 123 ± 279 mg/100 g serving and values ranged from 46 mg/100 g serving (pork sirloin roast) to 2193 mg/100 g serving (cured pork bacon). The sodium content of cooked (as prepared) plant-derived products was an average of 18 ± 21 mg/100 g serving, and values ranged from 1 mg/100 g serving (black beans) to 70 mg/100 g serving (spinach).

When discussing the importance of high-protein foods in terms of the amount of minerals provided, the two most important to discuss are generally thought to be iron and zinc. However, evaluation of mineral content alone does not fully characterize the nutritional contribution of a food, as differences in chemical form, absorption, and interactions with other dietary components influence nutrient utilization.

The chemical form of iron has a major impact on its absorption. The heme form of iron is better absorbed (approximately 15 to 40%) when compared with the non-heme form of iron (approximately 1 to 15%) [35]. The chemical form of iron in meat and fish is up to 40% heme, while plant-derived foods contain only non-heme iron [36]. Although the concentration of total iron may be greater in some plant-derived foods compared with animal-derived foods, the physiological availability of iron can be highly variable and is influenced by the chemical form of iron as well as other dietary components that promote or inhibit absorption.

The amount of zinc in animal-derived foods and plant-derived foods is fairly similar on an uncooked basis but is much greater in animal-derived foods compared with plant-derived foods on a cooked basis. Even while the total amount of zinc in meat-based diets can be similar to a well-planned plant-based diet, absorption of zinc in plant-based diets would likely be lower because of anti-nutritional components in plant-derived foods [35].

Anti-nutritional components of plant-derived foods can influence the digestibility and bioavailability of minerals. In particular, phytates, oxalates, and tannins are well-established antinutritional factors, while certain polyphenolic compounds may also reduce the absorption of minerals such as iron and zinc through complex formation [35,36].

#### 3.1.6. Protein:Calories

Protein:Calories, Protein:Total Fat and Saturated Fat:Total Fat of the food products in their uncooked and cooked/prepared state are presented in Table S6. The ratio of protein:calories for uncooked (as purchased) beef, pork, poultry, and seafood products was an average of 0.132 ± 0.047 g:kcal/100 g serving and values ranged from 0.030 g:kcal/100 g serving (cured pork bacon) to 0.224 g:kcal/100 g serving (yellowfin tuna). The ratio of protein:calories for uncooked (as purchased) plant-derived products was an average of 0.073 ± 0.032 g:kcal/100 g serving, and values ranged from 0.033 g:kcal/100 g serving (cashews) to 0.145 g:kcal/100 g serving (kidney beans).

The ratio of protein:calories for cooked (as prepared) beef, pork, poultry, and seafood products was an average of 0.138 ± 0.040 g:kcal/100 g serving and values ranged from 0.039 g:kcal/100 g serving (cured pork ham) to 0.224 g:kcal/100 g serving (yellowfin tuna). The ratio of protein:calories for cooked (as prepared) plant-derived products was an average of 0.070 ± 0.034 g:kcal/100 g serving and values ranged from 0.027 g:kcal/100 g serving (cashews) to 0.146 g:kcal/100 g serving (kidney beans).

To our knowledge, no previous descriptive analysis or review has evaluated the ratio of protein:calories or calories:protein. In this descriptive analysis, protein:calories represents the concentration of protein over calories, which means greater values indicate high protein foods with low calories and lower values indicate low protein foods with high calories. Beef, pork, poultry, and seafood products (on both an uncooked and cooked basis) were nearly two-fold greater on average in the ratio of protein:calories. Within the evaluated dataset, meat and seafood products generally provided greater concentrations of protein and lower calories per unit weight and cost compared with many plant-derived foods. However, nutrient density alone does not fully characterize the nutritional value of a food, as factors such as protein quality, amino acid composition, digestibility, bioavailability, dietary patterns, and health outcomes must also be considered.

#### 3.1.7. Protein:Total Fat

The ratio of protein:total fat for uncooked (as purchased) beef, pork, poultry, and seafood products was an average of 5.16 ± 7.95 g:g/100 g serving and values ranged from 0.32 g:g/100 g serving (cured pork bacon) to 49.80 g:g/100 g serving (yellowfin tuna). The ratio of protein:total fat for uncooked (as purchased) plant-derived products was an average of 7.83 ± 6.18 g:g/100 g serving and values ranged from 0.42 g:g/100 g serving (cashews and almonds) to 19.18 g:g/100 g serving (great northern beans).

The ratio of protein:total fat for cooked (as prepared) beef, pork, poultry, and seafood products was an average of 5.31 ± 7.86 g:g/100 g serving and values ranged from 0.43 g:g/100 g serving (cured pork ham) to 49.41 g:g/100 g serving (yellowfin tuna). The ratio of protein:total fat for cooked (as prepared) plant-derived products was an average of 9.98 ± 8.51 g:g/100 g serving and values ranged from 0.33 g:g/100 g serving (cashews) to 24.36 g:g/100 g serving (green peas).

To our knowledge, no previous descriptive analysis or review has evaluated the ratio of protein:total fat; however, the lean:fat is a common ratio used in processed meat products (e.g., 80/20 ground product). Similar to protein:calories, protein:total fat indicates the amount of protein over fat, hence greater values indicate foods with high protein and low fat and lower values indicate foods with low protein and high fat. Beef, pork, poultry, and seafood products had lower values for protein:total fat compared with plant-derived foods. There was a great amount of variation within beef, pork, poultry, and seafood products, indicating that there are options within animal-derived food products to achieve high protein and low fat.

#### 3.1.8. Protein:Saturated Fat

The ratio of protein:saturated fat for uncooked (as purchased) beef, pork, poultry, and seafood products was an average of 18.44 ± 34.34 g:g/100 g serving and values ranged from 0.95 g:g/100 g serving (cured pork bacon) to 179.34 g:g/100 g serving (haddock). The ratio of protein:saturated fat for uncooked (as purchased) plant-derived products was an average of 49.41 ± 39.69 g:g/100 g serving, and values ranged from 2.34 g:g/100 g serving (cashews) to 157.19 g:g/100 g serving (lentils).

The ratio of protein:saturated fat for cooked (as prepared) beef, pork, poultry, and seafood products was an average of 18.50 ± 34.44 g:g/100 g serving and values ranged from 1.20 g:g/100 g serving (cured pork ham) to 180.09 g:g/100 g serving (haddock). The ratio of protein:saturated fat for cooked (as prepared) plant-derived products was an average of 56.47 ± 48.98 g:g/100 g serving, and values ranged from 1.67 g:g/100 g serving (cashews) to 166.04 g:g/100 g serving (lentils).

Similar to protein:total fat, protein:saturated fat indicates the amount of protein over saturated fat, hence greater values indicate foods with high protein and low saturated fat and lower values indicate foods with low protein and high saturated fat. Unsurprisingly, beef, pork, poultry, and seafood products had much lower values for protein:saturated fat compared with plant-derived foods. There was a great amount of variation within beef, pork, poultry, and seafood products, indicating that there are options within animal-derived food products to achieve high protein and low fat.

#### 3.1.9. Saturated Fat:Total Fat

The ratio of saturated fat:total fat for uncooked (as purchased) beef, pork, poultry, and seafood products was an average of 0.34 ± 0.08 g:g/100 g serving and values ranged from 0.14 g:g/100 g serving (pollock) to 0.46 g:g/100 g serving (ground lamb). The ratio of saturated fat:total fat for uncooked (as purchased) plant-derived products was an average of 0.16 ± 0.07 g:g/100 g serving and values ranged from 0.08 g:g/100 g serving (almonds) to 0.31 g:g/100 g serving (great northern beans).

The ratio of saturated fat:total fat for cooked (as prepared) beef, pork, poultry, and seafood products was an average of 0.36 ± 0.08 g:g/100 g serving and values ranged from 0.13 g:g/100 g serving (pollock) to 0.46 g:g/100 g serving (beef eye of round steak and beef eye of round roast). The ratio of saturated fat:total fat for cooked (as prepared) plant-derived products was an average of 0.17 ± 0.06 g:g/100 g serving and values ranged from 0.08 g:g/100 g serving (almonds) to 0.31 g:g/100 g serving (great northern beans).

Saturated fat:total fat indicates the amount of saturated fat over fat, hence greater values indicate foods with high saturated fat content relative to total fat, and lower values indicate foods with low saturated fat relative to total fat. Unsurprisingly, beef, pork, poultry, and seafood products had much greater values for saturated fat:total fat compared with plant-derived foods. The magnitude of this difference was nearly a two-fold increase on an average basis.

#### 3.1.10. Summary for Nutrient Density

Although animal-derived foods often provide concentrated sources of highly digestible protein and several important micronutrients, plant-derived foods provide important nutritional and functional benefits that are not fully captured by nutrient density calculations alone. Many plant-derived foods contribute dietary fiber, phytochemicals, antioxidants, and bioactive compounds that may support gastrointestinal health and reduce risk factors associated with chronic diseases [37]. Dietary patterns emphasizing greater consumption of fruits, vegetables, legumes, whole grains, nuts, and seeds have been associated with improved cardiometabolic health outcomes [38]. Additionally, plant-derived protein sources can contribute meaningfully to protein intake, particularly when consumed in sufficient quantities or combined to complement limiting indispensable amino acids.

Beyond nutritional composition, environmental sustainability is an increasingly important consideration in evaluating food systems. Differences among protein sources in resource use, greenhouse gas emissions, land use, and production efficiency may influence dietary recommendations and consumer choices. Therefore, comparisons among protein foods should consider multiple dimensions, including nutrient composition, protein quality, affordability, health outcomes, cultural preferences, and environmental impacts.

The findings of this descriptive analysis should therefore be interpreted as a comparison of nutrient density among selected foods rather than a recommendation favoring one protein category over another. A balanced dietary approach that incorporates diverse protein sources may provide complementary nutritional benefits.

### 3.2. Nutrient Value (Cost of Nutrients) for Uncooked and Cooked Products

#### 3.2.1. Costs of Foods

Estimated retail cost of the food products are presented in Table S7. Retail cost of uncooked (as purchased) beef, pork, poultry, and seafood products was an average of 1.90 ± 0.98 Canadian dollars/100 g serving and values ranged from 0.22 Canadian dollars/100 g serving (turkey back) to 4.96 Canadian dollars/100 g serving (halibut). Retail cost of uncooked (as purchased) plant-derived products was an average of 1.22 ± 1.32 Canadian dollars/100 g serving, and values ranged from 0.32 kcal/100 g serving (lentils) to 4.32 Canadian dollars/100 g serving (cashews).

#### 3.2.2. Dietary Protein Value (i.e., Cost of Nutrients)

The estimated cost of nutrients are presented in Tables S8 and S9. For this descriptive analysis, only the cost of dietary protein is discussed because the estimated costs of other nutrients generally follow similar trends and therefore provide limited additional insight. Furthermore, the economic interpretation of individual nutrient costs is less intuitive and may not be directly meaningful. The dietary protein value of uncooked (as purchased) beef, pork, poultry, and seafood products was an average of 13.83 ± 10.52 g protein/Canadian dollar and values ranged from 3.74 g protein/Canadian dollar (halibut) to 81.93 g protein/Canadian dollar (turkey back). The dietary protein value of uncooked (as purchased) plant-derived products was an average of 18.00 ± 17.64 g protein/Canadian dollar, and values ranged from 1.34 g protein/Canadian dollar (kale) to 60.56 g protein/Canadian dollar (black beans).

The dietary protein value of cooked (as prepared) beef, pork, poultry, and seafood products was an average of 20.27 ± 16.42 g protein/Canadian dollar and values ranged from 4.40 g protein/Canadian dollar (chicken eggs) to 126.10 g protein/Canadian dollar (turkey back). The dietary protein value of cooked (as prepared) plant-derived products was an average of 11.55 ± 8.89 g protein/Canadian dollar, and values ranged from 0.60 g protein/Canadian dollar (kale) to 27.79 g protein/Canadian dollar (lentils).

On an uncooked basis, the protein value (amount of protein per dollar) was greater for plant-derived foods (average = 18.00 g protein/Canadian dollar) compared with beef, pork, poultry, and seafood foods (average = 13.83 g protein/Canadian dollar). Interestingly, on a cooked basis, the protein value (amount of protein per dollar) was greater for beef, pork, poultry, and seafood foods (average = 20.27 g protein/Canadian dollar) compared with plant-derived foods (average = 11.55 g protein/Canadian dollar). This is an interesting finding and establishes that the concept of the expense of foods should be considered on an “as prepared” basis that factors in individual nutrients.

## 4. Conclusions

This descriptive analysis provides Canadian consumers and the general population with a comparison of nutrient density and cost among high-protein foods. Meat and seafood products were consistent sources of protein and key nutrients, whereas many plant-derived foods evaluated contained lower protein concentrations on both an uncooked (as purchased) and cooked (as prepared) basis. Although individual foods varied considerably in vitamin, mineral, and nutrient cost profiles, meat products were not consistently more expensive when nutrient density was considered. These findings highlight the importance of evaluating food choices based on both nutrient contribution and cost rather than purchase price alone.

## Data Availability

No new data were created or analyzed in this study. Data sharing is not applicable to this article.

## Supplementary Materials

The following supporting information can be downloaded at: https://www.mdpi.com/article/10.3390/foods15152612/s1, Table S1: Dietary energy of different food products in their uncooked or cooked/prepared state (Source: Canadian Nutrient File Database); Table S2: Dietary protein of different food products in their uncooked or cooked/prepared state (Source: Canadian Nutrient File Database); Table S3: Total fat, saturated fat, and cholesterol of different food products in their uncooked or cooked/prepared state (Source: Canadian Nutrient File Database); Table S4: Vitamin B12 of different food products in their uncooked or cooked/prepared state (Source: Canadian Nutrient File Database); Table S5: Mineral content of different food products in their uncooked or cooked/prepared state (Source: Canadian Nutrient File Database); Table S6: Protein:Calories, Protein:Total Fat, Protein:Saturated Fat, and Saturated Fat:Total Fat of different food products in their uncooked or cooked/prepared state (Source: Canadian Nutrient File Database); Table S7: Estimated cost of foods; Table S8: Estimated cost of dietary energy, protein, total fat, saturated fat, and cholesterol nutrients for uncooked foods and cooked/prepared foods; Table S9: Estimated cost of vitamin B12 and minerals for uncooked foods and cooked/prepared foods.
