# Supplementary material for: A Descriptive Analysis of Nutrient Density and Nutritional Value of Meat Products Using the Canadian Nutrient File Database
Source: Foods. 2026 Jul 26;15(15):2612. doi: 10.3390/foods15152612 (PMC13465396; doi:10.3390/foods15152612)
Supplement: Supplementary file 1 [file foods-15-02612-s001.zip › foods-4429915-supplementary.pdf]

Table S1. Dietary energy of different food products in their uncooked or cooked/prepared state (Source: Canadian Nutrient File Database).

		Energy value (kcal)	
Product[1]	Food code	Uncooked	Cooked/Prepared
Meat			
Beef brisket	2676	155	218
Beef chuck blade roast	5946	147	254
Beef chuck cross rib roast	5943	151	240
Beef chuck short ribs	7360	235	305
Beef hip eye of round roast	5991	135	178
Beef hip eye of round steak	5997	131	207
Beef hip top round roast	6026	126	172
Beef hip top round steak	6032	120	219
Beef hip bottom round roast	6039	154	204
Beef hip bottom round steak	6045	153	238
Beef hip sirloin tip roast	6089	128	205
Beef hip sirloin tip steak	6095	130	220
Beef botom sirloin tri-tip roast	6147	166	242
Beef strip loin	6107	153	215
Beef t-bone steak	6115	182	247
Beef tenderloin	6123	159	206
Beef top sirloin	6137	132	178
Beef rib eye steak	6066	200	285
Beef rib eye roast	6061	239	296
Beef flank steak	6020	164	236
Beef rump roast	4757	184	218
Beef, ground extra lean	4996	158	222
Beef, ground lean	2683	207	258
Beef, ground medium	2690	226	285
Beef, ground regular	2786	293	324
Pork cured bacon	1936	417	548
Pork ground lean	6119	221	233
Pork ground medium	6121	270	299
Pork loin centre steak	1967	172	220
Pork loin centre chop	1781	170	242
Pork loin rib end	1789	186	261
Pork Country-style ribs	1905	189	273
Pork loin rib roast	6159	206	244
Pork sirloin chop	1797	168	234
Pork sirloin roast	1921	166	192
Pork tenderloin	1943	126	166
Pork side ribs centre cut	1933	256	334
Pork shoulder blade	1971	245	264

Pork shoulder picnic	1817	193	235
Pork cured ham	1868	315	342
Lamb foreshank	3428	111	195
Lamb leg	3432	113	221
Lamb loin	3445	120	200
Lamb rib	3451	129	206
Lamb shoulder	3458	118	234
Lamb ground	3639	204	205
Chicken back	832	319	300
Chicken breast	838	172	197
Chicken drumstick	844	161	191
Chicken thigh	850	221	232
Chicken wing	626	191	254
Chicken ground	918	143	189
Turkey back	712	168	240
Turkey breast	714	129	172
Turkey drumstick	6863	143	203
Turkey ground	815	146	196
Turkey thigh	6864	156	205
Turkey wing	718	168	220
Chicken egg, whole	125	145	141
Fish			
Catfish, wild channel	2993	95	105
Cod, Atlantic	3194	82	105
Haddock	3198	74	90
Halibut, Atlantic & Pacific	3200	91	111
Pollock, Atlantic	3038	92	118
Salmon, Atlantic, farmed	3182	208	206
Salmon, Atlantic, wild	3049	142	182
Sea bass	3056	97	124
Tilapia	5966	96	129
Trout	3204	148	190
Tuna, yellowfin	3085	109	130
Plant-derived foods			
Kale	2395	49	28
Broccoli	2374	34	35
Spinach	2213	23	23
Lentils, sprouted	2113	106	101
Green peas	2409	81	84
Black beans, mature	3376	341	132
Pinto beans, mature	2310	62	22
Lima beans	2003	113	123

Kidney beans, sprouted	2001	29	33
Great northern beans	3259	339	118
Peanuts	3396	568	587
Almonds	2534	579	598
Cashew nuts	5709	553	574
Quinoa	4495	368	120

[1]All products are standardized to a 100 g serving.

Table S2. Dietary protein of different food products in their uncooked or cooked/prepared state (Source: Canadian Nutrient File Database).

		Protein (g)	
Product[1]	Food code	Uncooked	Cooked/Prepared
Meat			
Beef brisket	2676	20.72	29.75
Beef chuck blade roast	5946	20.56	35.07
Beef chuck cross rib roast	5943	21.32	37.50
Beef chuck short ribs	7360	17.48	25.48
Beef hip eye of round roast	5991	22.83	32.89
Beef hip eye of round steak	5997	23.08	40.38
Beef hip top round roast	6026	22.56	31.92
Beef hip top round steak	6032	22.66	40.28
Beef hip bottom round roast	6039	22.32	34.02
Beef hip bottom round steak	6045	22.32	39.74
Beef hip sirloin tip roast	6089	21.70	33.12
Beef hip sirloin tip steak	6095	21.64	39.83
Beef botom sirloin tri-tip roast	6147	22.32	31.03
Beef strip loin	6107	21.87	32.22
Beef t-bone steak	6115	21.72	32.10
Beef tenderloin	6123	21.24	30.30
Beef top sirloin	6137	22.51	29.26
Beef rib eye steak	6066	21.18	27.78
Beef rib eye roast	6061	19.96	28.07
Beef flank steak	6020	21.67	34.41
Beef rump roast	4757	20.56	27.58
Beef, ground extra lean	4996	20.88	30.65
Beef, ground lean	2683	19.58	29.00
Beef, ground medium	2690	18.90	29.20
Beef, ground regular	2786	16.55	28.68
Pork cured bacon	1936	12.62	35.73
Pork ground lean	6119	18.80	25.77
Pork ground medium	6121	17.30	24.50
Pork loin centre steak	1967	22.05	31.95
Pork loin centre chop	1781	20.71	28.21
Pork loin rib end	1789	20.28	26.66
Pork Country-style ribs	1905	19.34	26.49
Pork loin rib roast	6159	20.48	28.22
Pork sirloin chop	1797	20.48	28.81
Pork sirloin roast	1921	21.34	26.45
Pork tenderloin	1943	22.10	28.56
Pork side ribs centre cut	1933	18.00	26.70

Pork shoulder blade	1971	17.36	25.20
Pork shoulder picnic	1817	18.71	24.88
Pork cured ham	1868	12.78	13.30
Lamb foreshank	3428	22.44	30.91
Lamb leg	3432	21.80	27.11
Lamb loin	3445	22.51	29.77
Lamb rib	3451	22.30	30.71
Lamb shoulder	3458	20.81	31.25
Lamb ground	3639	17.06	26.68
Chicken back	832	14.05	25.95
Chicken breast	838	20.85	29.80
Chicken drumstick	844	18.08	23.35
Chicken thigh	850	16.52	23.26
Chicken wing	626	17.52	23.79
Chicken ground	918	17.44	23.28
Turkey back	712	17.86	27.49
Turkey breast	714	22.83	31.10
Turkey drumstick	6863	19.06	30.02
Turkey ground	815	18.90	27.63
Turkey thigh	6864	18.83	27.72
Turkey wing	718	20.19	29.62
Chicken egg, whole	125	11.80	11.80
Fish			
Catfish, wild channel	2993	16.38	18.47
Cod, Atlantic	3194	17.81	22.83
Haddock	3198	16.32	19.99
Halibut, Atlantic & Pacific	3200	18.56	22.54
Pollock, Atlantic	3038	19.44	24.92
Salmon, Atlantic, farmed	3182	20.42	22.10
Salmon, Atlantic, wild	3049	19.84	25.44
Sea bass	3056	18.43	23.63
Tilapia	5966	20.08	26.15
Trout	3204	20.77	26.63
Tuna, yellowfin	3085	24.40	29.15
Plant-derived foods			
Kale	2395	4.28	1.90
Broccoli	2374	2.82	2.38
Spinach	2213	2.86	2.97
Lentils, sprouted	2113	8.96	8.80
Green peas	2409	5.42	5.36
Black beans, mature	3376	21.60	8.86
Pinto beans, mature	2310	5.25	1.86

Lima beans	2003	6.84	6.81
Kidney beans, sprouted	2001	4.20	4.83
Great northern beans	3259	21.86	8.33
Peanuts	3396	25.80	24.35
Almonds	2534	21.15	20.96
Cashew nuts	5709	18.22	15.31
Quinoa	4495	14.12	4.40

[1]All products are standardized to a 100 g serving.

Table S3. Total fat, saturated fat, and cholesterol of different food products in their uncooked or cooked/prepared state (Source: Canadian Nutrient File Database).

		Total Fat (g)		Saturated fat (g)		Cholesterol (mg)	
Product[1]	Food code	Uncooked	Cooked/Prepared	Uncooked	Cooked/Prepared	Uncooked	Cooked/Prepared
Meat							
Beef brisket	2676	7.37	10.08	2.590	3.630	62	93
Beef chuck blade roast	5946	6.73	11.55	2.690	4.680	58	95
Beef chuck cross rib roast	5943	6.58	8.50	2.670	3.510	51	93
Beef chuck short ribs	7360	18.33	22.58	7.979	6.300	75	100
Beef hip eye of round roast	5991	4.04	3.76	1.740	1.740	48	65
Beef hip eye of round steak	5997	3.45	3.23	1.450	1.500	48	84
Beef hip top round roast	6026	3.10	3.77	1.320	1.670	52	70
Beef hip top round steak	6032	2.54	5.03	1.070	2.100	51	91
Beef hip bottom round roast	6039	5.85	6.11	2.470	2.440	60	78
Beef hip bottom round steak	6045	5.69	7.03	2.390	2.740	60	97
Beef hip sirloin tip roast	6089	3.65	6.40	1.580	2.810	51	77
Beef hip sirloin tip steak	6095	3.87	4.81	1.690	2.070	51	93
Beef botom sirloin tri-tip roast	6147	7.59	11.96	3.200	4.970	53	74
Beef strip loin	6107	6.52	8.30	2.790	3.650	50	72
Beef t-bone steak	6115	10.03	12.34	4.180	5.250	51	70
Beef tenderloin	6123	7.48	8.19	3.200	3.680	55	69
Beef top sirloin	6137	3.97	5.94	1.620	2.410	53	73
Beef rib eye steak	6066	12.26	18.57	5.140	7.820	52	70
Beef rib eye roast	6061	17.01	19.29	7.090	8.390	55	71
Beef flank steak	6020	7.85	9.52	3.360	4.270	47	71
Beef rump roast	4757	10.68	11.08	4.200	4.230	62	79
Beef, ground extra lean	4996	7.59	10.12	3.132	4.205	55	78
Beef, ground lean	2683	13.68	14.85	5.462	5.968	60	79
Beef, ground medium	2690	16.10	17.82	6.670	7.422	60	82

Beef, ground regular	2786	24.70	22.32	10.168	9.302	66	84
Pork cured bacon	1936	39.69	43.27	13.296	14.187	66	107
Pork ground lean	6119	15.33	14.33	5.890	5.470	65	80
Pork ground medium	6121	21.70	21.50	8.210	8.020	67	88
Pork loin centre steak	1967	8.60	9.30	3.470	3.630	54	77
Pork loin centre chop	1781	9.03	13.51	2.993	4.934	69	81
Pork loin rib end	1789	11.04	16.28	2.365	5.435	58	79
Pork Country-style ribs	1905	11.82	17.71	2.370	6.302	74	103
Pork loin rib roast	6159	13.12	13.79	5.120	5.310	60	81
Pork sirloin chop	1797	8.96	12.31	1.851	4.294	70	87
Pork sirloin roast	1921	8.33	8.82	1.598	2.839	64	80
Pork tenderloin	1943	3.50	4.83	1.430	2.010	60	80
Pork side ribs centre cut	1933	19.90	24.40	7.560	9.350	67	99
Pork shoulder blade	1971	19.00	17.37	7.140	6.720	70	89
Pork shoulder picnic	1817	12.51	14.33	4.140	4.854	63	86
Pork cured ham	1868	28.19	30.85	10.130	11.090	70	72
Lamb foreshank	3428	2.35	7.92	0.878	3.128	69	70
Lamb leg	3432	2.70	12.52	1.090	5.593	72	70
Lamb loin	3445	3.29	8.96	1.382	3.791	72	70
Lamb rib	3451	4.38	9.19	1.877	4.036	72	70
Lamb shoulder	3458	3.71	12.13	1.503	5.339	72	70
Lamb ground	3639	14.12	10.94	6.426	4.736	65	70
Chicken back	832	28.74	20.97	8.340	5.827	79	88
Chicken breast	838	9.25	7.78	2.660	2.190	64	84
Chicken drumstick	844	9.20	10.15	2.459	2.740	92	130
Chicken thigh	850	16.61	14.71	4.524	4.111	98	133
Chicken wing	626	13.16	16.87	4.061	4.976	111	141
Chicken ground	918	8.10	10.92	2.301	3.110	86	107

Turkey back	712	10.22	13.56	3.214	4.384	84	136
Turkey breast	714	3.48	4.36	1.112	1.557	61	81
Turkey drumstick	6863	6.78	8.34	2.082	2.861	93	144
Turkey ground	815	7.23	8.71	2.168	2.863	87	127
Turkey thigh	6864	8.35	9.56	2.464	3.371	89	125
Turkey wing	718	9.48	10.36	2.755	3.808	77	121
Chicken egg, whole	125	10.01	10.01	3.043	3.043	366	366
Fish							
Catfish, wild channel	2993	2.82	2.85	0.722	0.744	58	72
Cod, Atlantic	3194	0.67	0.86	0.131	0.168	43	55
Haddock	3198	0.45	0.55	0.091	0.111	54	66
Halibut, Atlantic & Pacific	3200	1.33	1.61	0.292	0.354	49	60
Pollock, Atlantic	3038	0.98	1.26	0.135	0.170	71	91
Salmon, Atlantic, farmed	3182	13.42	12.35	3.050	2.504	55	63
Salmon, Atlantic, wild	3049	6.34	8.13	0.981	1.257	55	71
Sea bass	3056	2.00	2.56	0.511	0.655	41	53
Tilapia	5966	1.70	2.65	0.585	0.940	50	57
Trout	3204	6.61	8.47	1.149	1.474	58	74
Tuna, yellowfin	3085	0.49	0.59	0.172	0.205	39	47
Plant-derived foods							
Kale	2395	0.93	0.40	0.091	0.052	0	0
Broccoli	2374	0.37	0.41	0.039	0.079	0	0
Spinach	2213	0.39	0.26	0.063	0.043	0	0
Lentils, sprouted	2113	0.55	0.45	0.057	0.053	0	0
Green peas	2409	0.40	0.22	0.071	0.039	0	0
Black beans, mature	3376	1.42	0.54	0.366	0.139	0	0
Pinto beans, mature	2310	0.90	0.32	0.109	0.039	0	0
Lima beans	2003	0.86	0.32	0.198	0.073	0	0

Kidney beans, sprouted	2001	0.50	0.58	0.072	0.083	0	0
Great northern beans	3259	1.14	0.45	0.356	0.140	0	0
Peanuts	3396	49.24	49.66	6.279	7.723	0	0
Almonds	2534	49.93	52.54	3.802	4.092	0	0
Cashew nuts	5709	43.85	46.35	7.783	9.157	0	0
Quinoa	4495	6.07	1.92	0.706	0.231	0	0

[1]All products are standardized to a 100 g serving.

Table S4. Vitamin B12 of different food products in their uncooked or cooked/prepared state (Source: Canadian Nutrient File Database).

		Vitamin B12 (µg)	
Product[1]	Food code	Uncooked	Cooked/Prepared
Meat			
Beef brisket	2676	2.43	2.60
Beef chuck blade roast	5946	2.49	3.20
Beef chuck cross rib roast	5943	2.61	2.57
Beef chuck short ribs	7360	3.00	3.45
Beef hip eye of round roast	5991	1.58	1.97
Beef hip eye of round steak	5997	1.58	2.03
Beef hip top round roast	6026	1.95	2.24
Beef hip top round steak	6032	1.95	1.95
Beef hip bottom round roast	6039	2.05	2.26
Beef hip bottom round steak	6045	2.10	1.88
Beef hip sirloin tip roast	6089	1.89	2.51
Beef hip sirloin tip steak	6095	1.85	2.55
Beef botom sirloin tri-tip roast	6147	1.88	2.64
Beef strip loin	6107	1.75	2.44
Beef t-bone steak	6115	2.15	2.68
Beef tenderloin	6123	2.23	3.18
Beef top sirloin	6137	2.54	2.81
Beef rib eye steak	6066	1.67	2.10
Beef rib eye roast	6061	1.61	2.21
Beef flank steak	6020	2.51	2.91
Beef rump roast	4757	2.82	3.03
Beef, ground extra lean	4996	2.12	2.75
Beef, ground lean	2683	2.35	2.88
Beef, ground medium	2690	1.75	2.50
Beef, ground regular	2786	2.35	3.62
Pork cured bacon	1936	0.50	1.16
Pork ground lean	6119	0.77	1.06
Pork ground medium	6121	0.55	1.15
Pork loin centre steak	1967	0.50	0.76
Pork loin centre chop	1781	0.53	0.66
Pork loin rib end	1789	0.54	0.64
Pork Country-style ribs	1905	1.00	0.96
Pork loin rib roast	6159	0.91	1.07
Pork sirloin chop	1797	0.56	0.63
Pork sirloin roast	1921	0.54	0.58
Pork tenderloin	1943	0.52	0.98
Pork side ribs centre cut	1933	1.02	0.91
Pork shoulder blade	1971	1.03	1.23

Pork shoulder picnic	1817	0.57	0.74
Pork cured ham	1868	1.08	0.70
Lamb foreshank	3428	3.32	2.26
Lamb leg	3432	2.21	2.28
Lamb loin	3445	1.63	2.26
Lamb rib	3451	1.71	2.48
Lamb shoulder	3458	2.88	2.56
Lamb ground	3639	2.31	2.59
Chicken back	832	0.25	0.27
Chicken breast	838	0.34	0.32
Chicken drumstick	844	0.53	0.39
Chicken thigh	850	0.62	0.44
Chicken wing	626	0.25	0.35
Chicken ground	918	0.56	0.51
Turkey back	712	1.41	1.99
Turkey breast	714	1.76	0.96
Turkey drumstick	6863	1.76	2.19
Turkey ground	815	2.18	3.20
Turkey thigh	6864	1.50	1.88
Turkey wing	718	0.97	1.51
Chicken egg, whole	125	1.90	1.62
Fish			
Catfish, wild channel	2993	2.23	2.90
Cod, Atlantic	3194	0.91	1.05
Haddock	3198	1.83	2.13
Halibut, Atlantic & Pacific	3200	1.10	1.27
Pollock, Atlantic	3038	3.19	3.68
Salmon, Atlantic, farmed	3182	3.23	2.80
Salmon, Atlantic, wild	3049	3.18	3.05
Sea bass	3056	0.30	0.30
Tilapia	5966	1.58	1.86
Trout	3204	7.79	7.49
Tuna, yellowfin	3085	2.08	2.35
Plant-derived foods			
Kale	2395	0.00	0.00
Broccoli	2374	0.00	0.00
Spinach	2213	0.00	0.00
Lentils, sprouted	2113	0.00	0.00
Green peas	2409	0.00	0.00
Black beans, mature	3376	0.00	0.00
Pinto beans, mature	2310	0.00	0.00
Lima beans	2003	0.00	0.00

Kidney beans, sprouted	2001	0.00	0.00
Great northern beans	3259	0.00	0.00
Peanuts	3396	0.00	0.00
Almonds	2534	0.00	0.00
Cashew nuts	5709	0.00	0.00
Quinoa	4495	0.00	0.00

[1]All products are standardized to a 100 g serving.

Table S5. Mineral content of different food products in their uncooked or cooked/prepared state (Source: Canadian Nutrient File Database).

		Fe (mg)		P (mg)		Zn (mg)		Na (mg)	
Product[1]	Food code	Uncooked	Cooked/Prepared	Uncooked	Cooked/Prepared	Uncooked	Cooked/Prepared	Uncooked	Cooked/Prepared
Meat									
Beef brisket	2676	1.92	2.81	201	241	4.31	6.89	79	70
Beef chuck blade roast	5946	2.04	3.36	170	192	6.75	11.46	72	61
Beef chuck cross rib roast	5943	2.10	3.55	184	226	5.24	8.95	64	70
Beef chuck short ribs	7360	2.24	2.70	165	170	7.07	9.73	78	70
Beef hip eye of round roast	5991	1.55	2.02	221	279	4.25	5.96	59	54
Beef hip eye of round steak	5997	1.55	2.61	220	286	4.35	7.73	60	48
Beef hip top round roast	6026	2.10	2.85	195	218	5.28	7.19	61	65
Beef hip top round steak	6032	2.10	3.28	196	212	5.28	7.73	61	50
Beef hip bottom round roast	6039	2.05	3.88	214	202	3.94	7.37	70	56
Beef hip bottom round steak	6045	2.15	4.40	202	202	3.94	7.81	70	58
Beef hip sirloin tip roast	6089	2.15	3.55	202	202	5.30	8.46	59	59
Beef hip sirloin tip steak	6095	2.15	4.21	202	202	5.30	11.42	59	59
Beef botom sirloin tri-tip roast	6147	1.74	2.83	202	202	4.64	6.65	58	63
Beef strip loin	6107	2.15	2.94	200	265	4.30	7.01	58	59
Beef t-bone steak	6115	2.05	3.05	200	219	3.98	6.29	65	71
Beef tenderloin	6123	2.75	3.51	211	254	4.00	6.22	61	64
Beef top sirloin	6137	2.36	2.90	195	217	5.09	6.83	59	60
Beef rib eye steak	6066	2.23	3.30	202	202	5.34	7.74	55	54
Beef rib eye roast	6061	2.09	2.99	202	202	5.00	7.06	52	54
Beef flank steak	6020	1.80	2.90	202	202	6.15	10.28	63	61
Beef rump roast	4757	2.00	2.56	199	240	3.24	4.36	54	62
Beef, ground extra lean	4996	1.95	2.85	172	240	4.58	6.88	64	87
Beef, ground lean	2683	1.80	2.82	161	232	4.58	6.72	63	93
Beef, ground medium	2690	1.88	2.60	156	230	4.00	6.32	59	90

Beef, ground regular	2786	1.80	2.88	136	237	4.18	6.12	60	104
Pork cured bacon	1936	0.41	1.49	144	506	1.18	3.36	662	2193
Pork ground lean	6119	1.00	1.23	177	221	2.43	3.23	67	80
Pork ground medium	6121	0.75	1.10	155	218	2.10	2.90	60	90
Pork loin centre steak	1967	0.60	0.80	198	241	1.50	2.20	49	62
Pork loin centre chop	1781	0.63	0.86	209	225	1.77	3.28	55	73
Pork loin rib end	1789	0.59	0.81	203	213	1.85	3.10	56	70
Pork Country-style ribs	1905	0.85	1.34	193	202	2.78	4.23	63	58
Pork loin rib roast	6159	0.80	1.10	190	218	2.30	3.10	67	63
Pork sirloin chop	1797	0.82	0.71	209	285	1.95	1.97	57	58
Pork sirloin roast	1921	0.53	0.64	216	223	1.73	2.11	47	46
Pork tenderloin	1943	1.10	1.39	222	290	2.00	2.89	54	64
Pork side ribs centre cut	1933	0.88	1.40	147	143	2.80	4.50	92	65
Pork shoulder blade	1971	1.00	1.40	172	204	2.80	4.10	59	66
Pork shoulder picnic	1817	0.57	0.92	200	240	2.15	3.10	84	96
Pork cured ham	1868	1.05	1.61	149	101	1.57	1.90	1088	1063
Lamb foreshank	3428	1.30	1.80	187	175	5.95	8.66	95	137
Lamb leg	3432	1.33	1.78	193	201	3.84	4.67	68	216
Lamb loin	3445	1.31	1.72	190	206	3.19	4.94	83	78
Lamb rib	3451	1.20	1.86	181	226	3.80	4.13	88	113
Lamb shoulder	3458	1.13	1.88	184	213	4.77	5.27	78	107
Lamb ground	3639	1.02	1.52	157	217	3.41	6.60	68	83
Chicken back	832	0.94	1.42	113	154	1.26	2.25	64	87
Chicken breast	838	0.74	1.07	174	214	0.80	1.02	63	71
Chicken drumstick	844	0.71	1.11	163	195	1.86	2.36	106	123
Chicken thigh	850	0.68	1.08	157	216	1.29	1.73	81	102
Chicken wing	626	0.46	0.84	123	147	1.21	1.64	84	98
Chicken ground	918	0.82	0.93	178	234	1.47	1.92	60	75

Turkey back	712	0.48	0.65	147	166	1.98	2.53	68	85
Turkey breast	714	0.64	0.34	185	240	1.96	1.10	69	48
Turkey drumstick	6863	0.64	1.05	185	200	1.96	3.36	69	89
Turkey ground	815	0.97	1.08	211	298	2.44	3.25	70	84
Turkey thigh	6864	0.62	0.98	190	212	1.71	2.86	60	69
Turkey wing	718	0.30	0.50	158	182	1.44	2.15	61	77
Chicken egg, whole	125	1.39	1.39	126	126	1.16	1.16	125	125
Fish									
Catfish, wild channel	2993	0.30	0.35	209	304	0.51	0.61	43	50
Cod, Atlantic	3194	0.38	0.49	203	138	0.45	0.58	54	78
Haddock	3198	0.17	0.21	227	278	0.32	0.40	213	261
Halibut, Atlantic & Pacific	3200	0.16	0.20	236	287	0.36	0.43	68	82
Pollock, Atlantic	3038	0.46	0.59	221	283	0.47	0.60	86	110
Salmon, Atlantic, farmed	3182	0.34	0.34	240	252	0.36	0.43	59	61
Salmon, Atlantic, wild	3049	0.80	1.03	200	256	0.64	0.82	44	56
Sea bass	3056	0.29	0.37	194	248	0.40	0.52	68	87
Tilapia	5966	0.56	0.69	170	204	0.33	0.41	52	56
Trout	3204	1.50	1.92	245	314	0.66	0.85	52	67
Tuna, yellowfin	3085	0.77	0.92	278	333	0.37	0.45	45	54
Plant-derived foods									
Kale	2395	1.47	0.90	92	28	0.56	0.24	38	23
Broccoli	2374	0.73	0.67	66	67	0.41	0.45	33	41
Spinach	2213	2.71	3.57	49	56	0.53	0.76	79	70
Lentils, sprouted	2113	3.21	3.10	173	153	1.51	1.60	11	10
Green peas	2409	1.47	1.54	108	117	1.24	1.19	5	3
Black beans, mature	3376	5.02	2.10	352	140	3.65	1.12	5	1
Pinto beans, mature	2310	1.97	0.66	94	30	0.50	0.17	153	51
Lima beans	2003	3.14	2.45	136	130	0.78	0.79	8	17

Kidney beans, sprouted	2001	0.81	0.89	37	38	0.40	0.44	6	7
Great northern beans	3259	5.47	2.13	447	165	2.31	0.88	14	2
Peanuts	3396	4.58	1.58	376	363	3.27	2.77	18	6
Almonds	2534	3.71	3.73	481	471	3.12	3.31	1	3
Cashew nuts	5709	6.68	6.00	593	490	5.78	5.60	12	16
Quinoa	4495	4.57	1.49	457	152	3.10	1.09	5	7

[1]All products are standardized to a 100 g serving.

Table S6. Protein:Calories, Protein:Total Fat, Protein:Saturated Fat, and Saturated Fat:Total Fat of different food products in their uncooked or cooked/prepared state (Source: Canadian Nutrient File Database).

		Protein:Calories (g:kcal)		Protein:Total Fat (g:g)		Protein:Saturated Fat (g:g)		Saturated Fat:Total Fat (g:g)	
Product[1]	Food code	Uncooked	Cooked/Pre-pared	Uncooked	Cooked/Pre-pared	Uncooked	Cooked/Pre-pared	Uncooked	Cooked/Pre-pared
Meat									
Beef brisket	2676	0.134	0.136	2.81	2.95	8.00	8.20	0.35	0.36
Beef chuck blade roast	5946	0.140	0.138	3.05	3.04	7.64	7.49	0.40	0.41
Beef chuck cross rib roast	5943	0.141	0.156	3.24	4.41	7.99	10.68	0.41	0.41
Beef chuck short ribs	7360	0.074	0.084	0.95	1.13	2.19	4.04	0.44	0.28
Beef hip eye of round roast	5991	0.169	0.185	5.65	8.75	13.12	18.90	0.43	0.46
Beef hip eye of round steak	5997	0.176	0.195	6.69	12.50	15.92	26.92	0.42	0.46
Beef hip top round roast	6026	0.179	0.186	7.28	8.47	17.09	19.11	0.43	0.44
Beef hip top round steak	6032	0.189	0.184	8.92	8.01	21.18	19.18	0.42	0.42
Beef hip bottom round roast	6039	0.145	0.167	3.82	5.57	9.04	13.94	0.42	0.40
Beef hip bottom round steak	6045	0.146	0.167	3.92	5.65	9.34	14.50	0.42	0.39
Beef hip sirloin tip roast	6089	0.170	0.162	5.95	5.18	13.73	11.79	0.43	0.44
Beef hip sirloin tip steak	6095	0.166	0.181	5.59	8.28	12.80	19.24	0.44	0.43
Beef botom sirloin tri-tip roast	6147	0.134	0.128	2.94	2.59	6.98	6.24	0.42	0.42
Beef strip loin	6107	0.143	0.150	3.35	3.88	7.84	8.83	0.43	0.44
Beef t-bone steak	6115	0.119	0.130	2.17	2.60	5.20	6.11	0.42	0.43
Beef tenderloin	6123	0.134	0.147	2.84	3.70	6.64	8.23	0.43	0.45
Beef top sirloin	6137	0.171	0.164	5.67	4.93	13.90	12.14	0.41	0.41
Beef rib eye steak	6066	0.106	0.097	1.73	1.50	4.12	3.55	0.42	0.42
Beef rib eye roast	6061	0.084	0.095	1.17	1.46	2.82	3.35	0.42	0.43
Beef flank steak	6020	0.132	0.146	2.76	3.61	6.45	8.06	0.43	0.45
Beef rump roast	4757	0.112	0.127	1.93	2.49	4.90	6.52	0.39	0.38

Beef, ground extra lean	4996	0.132	0.138	2.75	3.03	6.67	7.29	0.41	0.42
Beef, ground lean	2683	0.095	0.112	1.43	1.95	3.58	4.86	0.40	0.40
Beef, ground medium	2690	0.084	0.102	1.17	1.64	2.83	3.93	0.41	0.42
Beef, ground regular	2786	0.056	0.089	0.67	1.28	1.63	3.08	0.41	0.42
Pork cured bacon	1936	0.030	0.065	0.32	0.83	0.95	2.52	0.33	0.33
Pork ground lean	6119	0.085	0.111	1.23	1.80	3.19	4.71	0.38	0.38
Pork ground medium	6121	0.064	0.082	0.80	1.14	2.11	3.05	0.38	0.37
Pork loin centre steak	1967	0.128	0.145	2.56	3.44	6.35	8.80	0.40	0.39
Pork loin centre chop	1781	0.122	0.117	2.29	2.09	6.92	5.72	0.33	0.37
Pork loin rib end	1789	0.109	0.102	1.84	1.64	8.58	4.91	0.21	0.33
Pork Country-style ribs	1905	0.102	0.097	1.64	1.50	8.16	4.20	0.20	0.36
Pork loin rib roast	6159	0.099	0.116	1.56	2.05	4.00	5.31	0.39	0.39
Pork sirloin chop	1797	0.122	0.123	2.29	2.34	11.06	6.71	0.21	0.35
Pork sirloin roast	1921	0.129	0.138	2.56	3.00	13.35	9.32	0.19	0.32
Pork tenderloin	1943	0.175	0.172	6.31	5.91	15.45	14.21	0.41	0.42
Pork side ribs centre cut	1933	0.070	0.080	0.90	1.09	2.38	2.86	0.38	0.38
Pork shoulder blade	1971	0.071	0.095	0.91	1.45	2.43	3.75	0.38	0.39
Pork shoulder picnic	1817	0.097	0.106	1.50	1.74	4.52	5.13	0.33	0.34
Pork cured ham	1868	0.041	0.039	0.45	0.43	1.26	1.20	0.36	0.36
Lamb foreshank	3428	0.202	0.159	9.55	3.90	25.56	9.88	0.37	0.39
Lamb leg	3432	0.193	0.123	8.07	2.17	20.00	4.85	0.40	0.45
Lamb loin	3445	0.188	0.149	6.84	3.32	16.29	7.85	0.42	0.42
Lamb rib	3451	0.173	0.149	5.09	3.34	11.88	7.61	0.43	0.44
Lamb shoulder	3458	0.176	0.134	5.61	2.58	13.85	5.85	0.41	0.44
Lamb ground	3639	0.084	0.130	1.21	2.44	2.65	5.63	0.46	0.43
Chicken back	832	0.044	0.087	0.49	1.24	1.68	4.45	0.29	0.28
Chicken breast	838	0.121	0.151	2.25	3.83	7.84	13.61	0.29	0.28
Chicken drumstick	844	0.112	0.122	1.97	2.30	7.35	8.52	0.27	0.27

Chicken thigh	850	0.075	0.100	0.99	1.58	3.65	5.66	0.27	0.28
Chicken wing	626	0.092	0.094	1.33	1.41	4.31	4.78	0.31	0.29
Chicken ground	918	0.122	0.123	2.15	2.13	7.58	7.49	0.28	0.28
Turkey back	712	0.106	0.115	1.75	2.03	5.56	6.27	0.31	0.32
Turkey breast	714	0.177	0.181	6.56	7.13	20.53	19.97	0.32	0.36
Turkey drumstick	6863	0.133	0.148	2.81	3.60	9.15	10.49	0.31	0.34
Turkey ground	815	0.129	0.141	2.61	3.17	8.72	9.65	0.30	0.33
Turkey thigh	6864	0.121	0.135	2.26	2.90	7.64	8.22	0.30	0.35
Turkey wing	718	0.120	0.135	2.13	2.86	7.33	7.78	0.29	0.37
Chicken egg, whole	125	0.081	0.084	1.18	1.18	3.88	3.88	0.30	0.30
Fish									
Catfish, wild channel	2993	0.172	0.176	5.81	6.48	22.69	24.83	0.26	0.26
Cod, Atlantic	3194	0.217	0.217	26.58	26.55	135.95	135.89	0.20	0.20
Haddock	3198	0.221	0.222	36.27	36.35	179.34	180.09	0.20	0.20
Halibut, Atlantic & Pacific	3200	0.204	0.203	13.95	14.00	63.56	63.67	0.22	0.22
Pollock, Atlantic	3038	0.211	0.211	19.84	19.78	144.00	146.59	0.14	0.13
Salmon, Atlantic, farmed	3182	0.098	0.107	1.52	1.79	6.70	8.83	0.23	0.20
Salmon, Atlantic, wild	3049	0.140	0.140	3.13	3.13	20.22	20.24	0.15	0.15
Sea bass	3056	0.190	0.191	9.22	9.23	36.07	36.08	0.26	0.26
Tilapia	5966	0.209	0.203	11.81	9.87	34.32	27.82	0.34	0.35
Trout	3204	0.140	0.140	3.14	3.14	18.08	18.07	0.17	0.17
Tuna, yellowfin	3085	0.224	0.224	49.80	49.41	141.86	142.20	0.35	0.35
Plant-derived foods									
Kale	2395	0.087	0.068	4.60	4.75	47.03	36.54	0.10	0.13
Broccoli	2374	0.083	0.068	7.62	5.80	72.31	30.13	0.11	0.19
Spinach	2213	0.124	0.129	7.33	11.42	45.40	69.07	0.16	0.17
Lentils, sprouted	2113	0.085	0.087	16.29	19.56	157.19	166.04	0.10	0.12
Green peas	2409	0.067	0.064	13.55	24.36	76.34	137.44	0.18	0.18

Black beans, mature	3376	0.063	0.067	15.21	16.41	59.02	63.74	0.26	0.26
Pinto beans, mature	2310	0.085	0.085	5.83	5.81	48.17	47.69	0.12	0.12
Lima beans	2003	0.061	0.055	7.95	21.28	34.55	93.29	0.23	0.23
Kidney beans, sprouted	2001	0.145	0.146	8.40	8.33	58.33	58.19	0.14	0.14
Great northern beans	3259	0.064	0.071	19.18	18.51	61.40	59.50	0.31	0.31
Peanuts	3396	0.045	0.041	0.52	0.49	4.11	3.15	0.13	0.16
Almonds	2534	0.037	0.035	0.42	0.40	5.56	5.12	0.08	0.08
Cashew nuts	5709	0.033	0.027	0.42	0.33	2.34	1.67	0.18	0.20
Quinoa	4495	0.038	0.037	2.33	2.29	20.00	19.05	0.12	0.12

[1]All products are standardized to a 100 g serving.

Table S7. Estimated cost of foods.

Product[1]		Estimated Retail Cost/100 g (CAD$/100 g)[2]
Meat		
	Beef brisket	-
	Beef blade roast	1.82
	Beef chuck cross rib roast	1.76
	Beef chuck short ribs	2.27
	Beef hip eye of round roast	1.77
	Beef hip eye of round steak	1.95
	Beef hip top round roast	1.81
	Beef hip top round steak	2.13
	Beef hip bottom round roast	1.72
	Beef hip bottom round steak	1.85
	Beef hip sirloin tip roast	1.64
	Beef hip sirloin tip steak	1.85
	Beef bottom sirloin tri-tip roast	2.20
	Beef strip loin	3.26
	Beef t-bone steak	3.43
	Beef tenderloin	4.87
	Beef top sirloin	2.46
	Beef rib eye steak	3.47
	Beef rib eye roast	-
	Beef flank steak	2.78
	Beef rump roast	2.03
	Beef, ground extra lean	1.49
	Beef, ground lean	1.27
	Beef, ground medium	1.06
	Beef, ground regular	0.99
	Pork cured bacon	1.15
	Pork ground lean	1.00
	Pork ground medium	-
	Pork loin centre steak	-
	Pork loin centre chop	1.41
	Pork loin rib end	1.20
	Pork Country-style ribs	1.28
	Pork loin rib roast	1.18
	Pork sirloin chop	1.31
	Pork sirloin roast	1.37
	Pork tenderloin	1.23
	Pork side ribs center cut	1.35
	Pork shoulder blade	1.00

	Pork shoulder picnic	0.53
	Pork cured ham	1.54
	Lamb foreshank	2.42
	Lamb leg	2.15
	Lamb loin	3.35
	Lamb rib	4.13
	Lamb shoulder	2.58
	Lamb ground	2.32
	Chicken back	0.47
	Chicken breast	1.12
	Chicken drumstick	0.84
	Chicken thigh	0.83
	Chicken wing	1.12
	Chicken ground	1.26
	Turkey back	0.22
	Turkey breast	1.57
	Turkey drumstick	2.22
	Turkey ground	1.31
	Turkey thigh	0.91
	Turkey wing	0.77
	Chicken egg, whole	2.68
Fish		
	Catfish, wild channel	-
	Cod, Atlantic	2.63
	Haddock	2.66
	Halibut, Atlantic & Pacific	4.96
	Pollock, Atlantic	1.12
	Salmon, Atlantic, farmed	3.04
	Salmon, Atlantic, wild	-
	Sea bass	2.33
	Tilapia	1.91
	Trout	2.61
	Tuna, yellowfin	2.56
Plant-derived foods		
	Kale	3.18
	Broccoli	0.64
	Spinach	0.99
	Lentils, sprouted	0.32
	Green peas	0.34
	Black beans, mature	0.36
	Pinto beans, mature	0.37
	Lima beans	0.66

Kidney beans, sprouted	0.32
Great northern beans	0.44
Peanuts	1.19
Almonds	3.18
Cashew nuts	4.32
Quinoa	0.77

[1]All products are standardized to a 100 g serving.

[2]Source of estimation: marketplace assessment was conducted at three different retailers on 9-Dec-2019, 15-Jan-2020, 15-Feb-2020, 15-Mar-2020, and 15-April-2020.

Table S8. Estimated cost of dietary energy, protein, total fat, saturated fat, and cholesterol nutrients for uncooked foods and cooked/prepared foods.

	Energy value (kcal)		Protein (g/$)		Total Fat (g/$)		Saturated fat (g/$)		Cholesterol (mg/$)	
Product[1,2]	Uncooked	Cooked/Prepared	Uncooked	Cooked/Prepared	Uncooked	Cooked/Prepared	Uncooked	Cooked/Prepared	Uncooked	Cooked/Prepared
Meat										
Beef brisket	-	-	-	-	-	-	-	-	-	-
Beef blade roast	80.73	139.50	11.29	19.26	3.70	6.34	1.48	2.57	31.85	52.18
Beef chuck cross rib roast	85.75	136.29	12.11	21.29	3.74	4.83	1.52	1.99	28.96	52.81
Beef chuck short ribs	103.58	134.44	7.70	11.23	8.08	9.95	3.52	2.78	33.06	44.08
Beef hip eye of round roast	76.31	100.62	12.91	18.59	2.28	2.13	0.98	0.98	27.13	36.74
Beef hip eye of round steak	67.02	105.91	11.81	20.66	1.77	1.65	0.74	0.77	24.56	42.98
Beef hip top round roast	69.78	95.26	12.49	17.68	1.72	2.09	0.73	0.92	28.80	38.77
Beef hip top round steak	56.34	102.82	10.64	18.91	1.19	2.36	0.50	0.99	23.94	42.72
Beef hip bottom round roast	89.49	118.54	12.97	19.77	3.40	3.55	1.44	1.42	34.87	45.33
Beef hip bottom round steak	82.70	128.65	12.06	21.48	3.08	3.80	1.29	1.48	32.43	52.43
Beef hip sirloin tip roast	78.02	124.95	13.23	20.19	2.22	3.90	0.96	1.71	31.09	46.93
Beef hip sirloin tip steak	70.41	119.15	11.72	21.57	2.10	2.61	0.92	1.12	27.62	50.37
Beef bottom sirloin tri-tip roast	75.56	110.15	10.16	14.12	3.45	5.44	1.46	2.26	24.12	33.68
Beef strip loin	46.92	65.93	6.71	9.88	2.00	2.55	0.86	1.12	15.33	22.08
Beef t-bone steak	52.99	71.91	6.32	9.35	2.92	3.59	1.22	1.53	14.85	20.38
Beef tenderloin	32.65	42.30	4.36	6.22	1.54	1.68	0.66	0.76	11.29	14.17
Beef top sirloin	53.75	72.48	9.17	11.91	1.62	2.42	0.66	0.98	21.58	29.72
Beef rib eye steak	57.67	82.18	6.11	8.01	3.54	5.35	1.48	2.26	15.00	20.19
Beef rib eye roast	-	.	-	.	-	.	-	.	-	.
Beef flank steak	59.08	85.02	7.81	12.40	2.83	3.43	1.21	1.54	16.93	25.58
Beef rump roast	90.82	107.60	10.15	13.61	5.27	5.47	2.07	2.09	30.60	38.99
Beef, ground extra lean	106.17	149.18	14.03	20.60	5.10	6.80	2.10	2.83	36.96	52.41
Beef, ground lean	163.33	203.57	15.45	22.88	10.79	11.72	4.31	4.71	47.34	62.33

Beef, ground medium	213.73	269.52	17.87	27.61	15.23	16.85	6.31	7.02	56.74	77.55
Beef, ground regular	295.96	327.27	16.72	28.97	24.95	22.55	10.27	9.40	66.67	84.85
Pork cured bacon	363.19	477.28	10.99	31.12	34.57	37.69	11.58	12.36	57.48	93.19
Pork ground lean	221.25	233.26	18.82	25.80	15.35	14.35	5.90	5.48	65.07	80.09
Pork ground medium	-	-	-	-	-	-	-	-	-	-
Pork loin centre steak	-	-	-	-	-	-	-	-	-	-
Pork loin centre chop	120.58	171.66	14.69	20.01	6.41	9.58	2.12	3.50	48.94	57.45
Pork loin rib end	155.08	217.61	16.91	22.23	9.20	13.57	1.97	4.53	48.36	65.87
Pork Country-style ribs	148.24	214.12	15.17	20.78	9.27	13.89	1.86	4.94	58.04	80.78
Pork loin rib roast	174.96	207.24	17.39	23.97	11.14	11.71	4.35	4.51	50.96	68.80
Pork sirloin chop	127.78	177.98	15.58	21.91	6.81	9.36	1.41	3.27	53.24	66.17
Pork sirloin roast	120.94	139.88	15.55	19.27	6.07	6.43	1.16	2.07	46.63	58.28
Pork tenderloin	102.48	135.01	17.97	23.23	2.85	3.93	1.16	1.63	48.80	65.07
Pork side ribs center cut	189.14	246.77	13.30	19.73	14.70	18.03	5.59	6.91	49.50	73.14
Pork shoulder blade	246.06	265.14	17.43	25.31	19.08	17.45	7.17	6.75	70.30	89.38
Pork shoulder picnic	366.22	445.92	35.50	47.21	23.74	27.19	7.86	9.21	119.54	163.19
Pork cured ham	205.14	222.72	8.32	8.66	18.36	20.09	6.60	7.22	45.59	46.89
Lamb foreshank	45.81	80.48	9.26	12.76	0.97	3.27	0.36	1.29	28.48	28.89
Lamb leg	52.63	102.93	10.15	12.63	1.26	5.83	0.51	2.61	33.54	32.60
Lamb loin	35.77	59.62	6.71	8.87	0.98	2.67	0.41	1.13	21.46	20.87
Lamb rib	31.26	49.91	5.40	7.44	1.06	2.23	0.45	0.98	17.44	16.96
Lamb shoulder	45.81	90.83	8.08	12.13	1.44	4.71	0.58	2.07	27.95	27.17
Lamb ground	88.06	88.50	7.36	11.52	6.10	4.72	2.77	2.04	28.06	30.22
Chicken back	675.85	635.59	29.77	54.98	60.89	44.43	17.67	12.35	167.37	186.44
Chicken breast	152.95	175.18	18.54	26.50	8.23	6.92	2.37	1.95	56.91	74.70
Chicken drumstick	191.16	226.78	21.47	27.72	10.92	12.05	2.92	3.25	109.23	154.35
Chicken thigh	266.74	280.01	19.94	28.07	20.05	17.75	5.46	4.96	118.28	160.52
Chicken wing	170.63	226.91	15.65	21.25	11.76	15.07	3.63	4.45	99.16	125.96

Chicken ground	113.13	149.53	13.80	18.42	6.41	8.64	1.82	2.46	68.04	84.65
Turkey back	770.64	1100.92	81.93	126.10	46.88	62.20	14.74	20.11	385.32	623.85
Turkey breast	82.15	109.53	14.54	19.80	2.22	2.78	0.71	0.99	38.85	51.58
Turkey drumstick	64.52	91.59	8.60	13.54	3.06	3.76	0.94	1.29	41.96	64.97
Turkey ground	111.58	149.79	14.44	21.12	5.53	6.66	1.66	2.19	66.49	97.06
Turkey thigh	170.96	224.66	20.64	30.38	9.15	10.48	2.70	3.69	97.53	136.99
Turkey wing	218.32	285.90	26.24	38.49	12.32	13.46	3.58	4.95	100.06	157.24
Chicken egg, whole	54.01	52.52	4.40	4.40	3.73	3.73	1.13	1.13	136.33	136.33
Fish										
Catfish, wild channel	-	-	-	-	-	-	-	-	-	-
Cod, Atlantic	31.21	39.97	6.78	8.69	0.26	0.33	0.05	0.06	16.37	20.93
Haddock	27.80	33.82	6.13	7.51	0.17	0.21	0.03	0.04	20.29	24.80
Halibut, Atlantic & Pacific	18.35	22.38	3.74	4.54	0.27	0.32	0.06	0.07	9.88	12.10
Pollock, Atlantic	82.14	105.36	17.36	22.25	0.88	1.13	0.12	0.15	63.39	81.25
Salmon, Atlantic, farmed	68.33	67.67	6.71	7.26	4.41	4.06	1.00	0.82	18.07	20.69
Salmon, Atlantic, wild	-	-	-	-	-	-	-	-	-	-
Sea bass	41.55	53.11	7.89	10.12	0.86	1.10	0.22	0.28	17.56	22.70
Tilapia	50.15	67.39	10.49	13.66	0.89	1.38	0.31	0.49	26.12	29.78
Trout	56.65	72.73	7.95	10.19	2.53	3.24	0.44	0.56	22.20	28.32
Tuna, yellowfin	42.63	50.84	9.54	11.40	0.19	0.23	0.07	0.08	15.25	18.38
Plant-derived foods										
Kale	15.39	8.80	1.34	0.60	0.29	0.13	0.03	0.02	0.00	0.00
Broccoli	53.13	54.70	4.41	3.72	0.58	0.64	0.06	0.12	0.00	0.00
Spinach	23.12	23.12	2.87	2.99	0.39	0.26	0.06	0.04	0.00	0.00
Lentils, sprouted	334.74	318.95	28.29	27.79	1.74	1.42	0.18	0.17	0.00	0.00
Green peas	240.36	249.26	16.08	15.91	1.19	0.65	0.21	0.12	0.00	0.00
Black beans, mature	956.07	370.09	60.56	24.84	3.98	1.51	1.03	0.39	0.00	0.00
Pinto beans, mature	168.48	59.78	14.27	5.05	2.45	0.87	0.30	0.11	0.00	0.00

Lima beans	171.21	186.36	10.36	10.32	1.30	0.48	0.30	0.11	0.00	0.00
Kidney beans, sprouted	91.19	103.77	13.21	15.19	1.57	1.82	0.23	0.26	0.00	0.00
Great northern beans	770.45	268.18	49.68	18.93	2.59	1.02	0.81	0.32	0.00	0.00
Peanuts	477.31	493.28	21.68	20.46	41.38	41.73	5.28	6.49	0.00	0.00
Almonds	181.85	187.81	6.64	6.58	15.68	16.50	1.19	1.29	0.00	0.00
Cashew nuts	127.97	132.83	4.22	3.54	10.15	10.73	1.80	2.12	0.00	0.00
Quinoa	479.08	156.22	18.38	5.73	7.90	2.50	0.92	0.30	0.00	0.00

[1]All products are standardized to a 100 g serving.

[2]Source of estimation: marketplace assessment was conducted at three different retailers on 9-Dec-2019, 15-Jan-2020, 15-Feb-2020, 15-Mar-2020, and 15-April-2020.

Table S9. Estimated cost of vitamin B12 and minerals for uncooked foods and cooked/prepared foods.

	Vitamin B12 (µg/$)		Fe (mg/$)		P (mg/$)		Zn (mg/$)		Na (mg/$)	
Product[1,2]	Uncooked	Cooked/Pre-pared	Uncooked	Cooked/Pre-pared	Uncooked	Cooked/Pre-pared	Uncooked	Cooked/Pre-pared	Uncooked	Cooked/Pre-pared
Meat										
Beef brisket	-	-	-	-	-	-	-	-	-	-
Beef blade roast	1.37	1.76	1.12	1.85	93.37	105.45	3.71	6.29	39.54	33.50
Beef chuck cross rib roast	1.48	1.46	1.19	2.02	104.49	128.34	2.98	5.08	36.34	39.75
Beef chuck short ribs	1.32	1.52	0.99	1.19	72.73	74.93	3.12	4.29	34.38	30.85
Beef hip eye of round roast	0.89	1.11	0.88	1.14	124.93	157.72	2.40	3.37	33.35	30.53
Beef hip eye of round steak	0.81	1.04	0.79	1.34	112.56	146.33	2.23	3.95	30.70	24.56
Beef hip top round roast	1.08	1.24	1.16	1.58	108.00	120.74	2.92	3.98	33.78	36.00
Beef hip top round steak	0.92	0.92	0.99	1.54	92.02	99.53	2.48	3.63	28.64	23.47
Beef hip bottom round roast	1.19	1.31	1.19	2.25	124.36	117.38	2.29	4.28	40.68	32.54
Beef hip bottom round steak	1.14	1.02	1.16	2.38	109.19	109.19	2.13	4.22	37.84	31.35
Beef hip sirloin tip roast	1.15	1.53	1.31	2.16	123.12	123.12	3.23	5.16	35.96	35.96
Beef hip sirloin tip steak	1.00	1.38	1.16	2.28	109.40	109.40	2.87	6.18	31.95	31.95
Beef bottom sirloin tri-tip roast	0.86	1.20	0.79	1.29	91.94	91.94	2.11	3.03	26.40	28.68
Beef strip loin	0.54	0.75	0.66	0.90	61.33	81.26	1.32	2.15	17.79	18.09
Beef t-bone steak	0.63	0.78	0.60	0.89	58.23	63.76	1.16	1.83	18.92	20.67
Beef tenderloin	0.46	0.65	0.56	0.72	43.33	52.16	0.82	1.28	12.53	13.14
Beef top sirloin	1.03	1.14	0.96	1.18	79.40	88.36	2.07	2.78	24.02	24.43
Beef rib eye steak	0.48	0.61	0.64	0.95	58.25	58.25	1.54	2.23	15.86	15.57
Beef rib eye roast	-	-	-	-	-	-	-	-	-	-
Beef flank steak	0.90	1.05	0.65	1.04	72.77	72.77	2.22	3.70	22.70	21.98
Beef rump roast	1.39	1.50	0.99	1.26	98.22	118.46	1.60	2.15	26.65	30.60
Beef, ground extra lean	1.42	1.85	1.31	1.92	115.58	161.27	3.08	4.62	43.01	58.46
Beef, ground lean	1.85	2.27	1.42	2.23	127.03	183.05	3.61	5.30	49.71	73.38

Beef, ground medium	1.65	2.36	1.78	2.46	147.53	217.51	3.78	5.98	55.80	85.11
Beef, ground regular	2.37	3.66	1.82	2.91	137.37	239.39	4.22	6.18	60.61	105.05
Pork cured bacon	0.44	1.01	0.36	1.30	125.42	440.70	1.03	2.93	576.57	1910.00
Pork ground lean	0.77	1.06	1.00	1.23	177.20	221.25	2.43	3.23	67.08	80.09
Pork ground medium	-	-	-	-	-	-	-	-	-	-
Pork loin centre steak	-	-	-	-	-	-	-	-	-	-
Pork loin centre chop	0.38	0.47	0.45	0.61	148.25	159.60	1.26	2.33	39.01	51.78
Pork loin rib end	0.45	0.53	0.49	0.68	169.25	177.59	1.54	2.58	46.69	58.36
Pork Country-style ribs	0.78	0.75	0.67	1.05	151.37	158.43	2.18	3.32	49.41	45.49
Pork loin rib roast	0.77	0.91	0.68	0.93	161.37	185.15	1.95	2.63	56.91	53.51
Pork sirloin chop	0.43	0.48	0.62	0.54	158.96	216.77	1.48	1.50	43.35	44.11
Pork sirloin roast	0.39	0.42	0.39	0.47	157.37	162.47	1.26	1.54	34.24	33.51
Pork tenderloin	0.42	0.80	0.89	1.13	180.56	235.86	1.63	2.35	43.92	52.05
Pork side ribs center cut	0.75	0.67	0.65	1.03	108.61	105.65	2.07	3.32	67.97	48.02
Pork shoulder blade	1.03	1.24	1.00	1.41	172.74	204.88	2.81	4.12	59.25	66.29
Pork shoulder picnic	1.08	1.40	1.08	1.75	379.51	455.41	4.08	5.88	159.39	182.16
Pork cured ham	0.70	0.46	0.68	1.05	97.03	65.77	1.02	1.24	708.54	692.26
Lamb foreshank	1.37	0.93	0.54	0.74	77.18	72.22	2.46	3.57	39.21	56.54
Lamb leg	1.03	2.46	0.62	0.83	89.89	93.62	1.79	2.18	31.67	100.61
Lamb loin	0.49	0.67	0.39	0.51	56.64	61.40	0.95	1.47	24.74	23.25
Lamb rib	0.41	0.60	0.29	0.45	43.85	54.76	0.92	1.00	21.32	27.38
Lamb shoulder	1.12	0.99	0.44	0.73	71.43	82.68	1.85	2.05	30.28	41.54
Lamb ground	1.00	1.12	0.44	0.66	67.77	93.68	1.47	2.85	29.35	35.83
Chicken back	0.53	0.57	1.99	3.01	239.41	326.27	2.67	4.77	135.59	184.32
Chicken breast	0.30	0.28	0.66	0.95	154.73	190.30	0.71	0.91	56.02	63.14
Chicken drumstick	0.63	0.46	0.84	1.32	193.53	231.53	2.21	2.80	125.86	146.04
Chicken thigh	0.75	0.53	0.82	1.30	189.49	260.70	1.56	2.09	97.76	123.11
Chicken wing	0.22	0.31	0.41	0.75	109.88	131.32	1.08	1.47	75.04	87.55

Chicken ground	0.44	0.40	0.65	0.74	140.82	185.13	1.16	1.52	47.47	59.34
Turkey back	6.47	9.13	2.20	2.98	674.31	761.47	9.08	11.61	311.93	389.91
Turkey breast	1.12	0.61	0.41	0.22	117.81	152.83	1.25	0.70	43.94	30.57
Turkey drumstick	0.79	0.99	0.29	0.47	83.46	90.23	0.88	1.52	31.13	40.15
Turkey ground	1.67	2.45	0.74	0.83	161.25	227.74	1.86	2.48	53.50	64.19
Turkey thigh	1.64	2.06	0.68	1.07	208.22	232.33	1.87	3.13	65.75	75.62
Turkey wing	1.26	1.96	0.39	0.65	205.33	236.52	1.87	2.79	79.27	100.06
Chicken egg, whole	0.71	0.60	0.52	0.52	46.93	46.93	0.43	0.43	46.56	46.56
Fish										
Catfish, wild channel	-	-	-	-	-	-	-	-	-	-
Cod, Atlantic	0.35	0.40	0.14	0.19	77.27	52.53	0.17	0.22	20.55	29.69
Haddock	0.69	0.80	0.06	0.08	85.29	104.45	0.12	0.15	80.03	98.06
Halibut, Atlantic & Pacific	0.22	0.26	0.03	0.04	47.58	57.86	0.07	0.09	13.71	16.53
Pollock, Atlantic	2.85	3.29	0.41	0.53	197.32	252.68	0.42	0.54	76.79	98.21
Salmon, Atlantic, farmed	1.06	0.92	0.11	0.11	78.84	82.78	0.12	0.14	19.38	20.04
Salmon, Atlantic, wild	-	-	-	-	-	-	-	-	-	-
Sea bass	0.13	0.13	0.12	0.16	83.09	106.22	0.17	0.22	29.12	37.26
Tilapia	0.83	0.97	0.29	0.36	88.80	106.56	0.17	0.21	27.16	29.25
Trout	2.98	2.87	0.57	0.73	93.78	120.19	0.25	0.33	19.90	25.65
Tuna, yellowfin	0.81	0.92	0.30	0.36	108.71	130.22	0.14	0.18	17.60	21.12
Plant-derived foods										
Kale	0.00	0.00	0.46	0.28	28.90	8.80	0.18	0.08	11.94	7.23
Broccoli	0.00	0.00	1.14	1.05	103.14	104.71	0.64	0.70	51.57	64.07
Spinach	0.00	0.00	2.72	3.59	49.26	56.29	0.53	0.76	79.41	70.37
Lentils, sprouted	0.00	0.00	10.14	9.79	546.32	483.16	4.77	5.05	34.74	31.58
Green peas	0.00	0.00	4.36	4.57	320.47	347.18	3.68	3.53	14.84	8.90
Black beans, mature	0.00	0.00	14.07	5.89	986.92	392.52	10.23	3.14	14.02	2.80
Pinto beans, mature	0.00	0.00	5.35	1.79	255.43	81.52	1.36	0.46	415.76	138.59

Lima beans	0.00	0.00	4.76	3.71	206.06	196.97	1.18	1.20	12.12	25.76
Kidney beans, sprouted	0.00	0.00	2.55	2.80	116.35	119.50	1.26	1.38	18.87	22.01
Great northern beans	0.00	0.00	12.43	4.84	1015.91	375.00	5.25	2.00	31.82	4.55
Peanuts	0.00	0.00	3.85	1.33	315.97	305.04	2.75	2.33	15.13	5.04
Almonds	0.00	0.00	1.17	1.17	151.07	147.93	0.98	1.04	0.31	0.94
Cashew nuts	0.00	0.00	1.55	1.39	137.23	113.39	1.34	1.30	2.78	3.70
Quinoa	0.00	0.00	5.95	1.94	594.94	197.88	4.04	1.42	6.51	9.11

[1]All products are standardized to a 100 g serving.

[2]Source of estimation: marketplace assessment was conducted at three different retailers on 9-Dec-2019, 15-Jan-2020, 15-Feb-2020, 15-Mar-2020, and 15-April-2020.
